# Experiencing sexual harassment by males and associated substance use & poor mental health outcomes among adolescent girls in the US

**DOI:** 10.1016/j.ssmph.2019.100476

**Published:** 2019-11-20

**Authors:** Elizabeth Reed, Marissa Salazar, Niloufar Agah, Alma I. Behar, Jay G Silverman, Eric Walsh-Buhi, Melanie L.A. Rusch, Anita Raj

**Affiliations:** a San Diego State University, School of Public Health, 5500 Campanile Dr, San Diego, CA, 92128, USA; b University of California San Diego, Center on Gender Equity and Health, Division of Global Public Health, 9500 Gilman Dr, La Jolla, CA, 92093, USA

**Keywords:** Adolescents, Sexual harassment, Substance use, Depression, Anxiety, Mental health

## Abstract

**Purpose:**

Among a sample of adolescent girls, we assessed: 1) prevalence of sexual harassment by type, place of occurrence, and perpetrators; 2) association with substance use and poor mental health outcomes; and 3) the proportion of girls experiencing sexual harassment in *more than one place* and relation to study outcomes.

**Methods:**

We collected survey data from 159 sexually active girls (aged 15–19 years) recruited from an urban health clinic in Southern California. We used multivariable regression models to assess associations between sexual harassment in the past 6 months and the following outcomes: 1) substance use in past 30 days (alcohol use, binge drinking, and marijuana use, respectively); 2) other drug use ever; and 3) feelings of depression and anxiety, respectively, in past 30 days.

**Results:**

Almost two-thirds of girls (65.4%) reported experiencing sexual harassment in the past 6 months. Among those reporting recent harassment (n = 104), the abuse most frequently occurred in public spaces (on public transport [50.5%], at school [42.7%], and in their neighborhoods [39.8%]) and most often in the form of sexual comments, jokes, or gestures (57.2%), although 12.6% were “touched, grabbed, or pinched in a sexual way.” The vast majority (82.7%) did not know the perpetrators (82.7%). Experiences of sexual harassment in the past 6 months were significantly associated with alcohol use (odds ratio [OR], 4.5; 95% confidence interval [CI], 2.0–10.2), binge drinking (OR, 4.2; 95% CI, 1.8–9.8), and marijuana use (OR, 2.6; 95% CI, 1.2–5.7) in the past 30 days; other drug use ever (OR, 5.4; 95% CI, 1.8–16.4); and feelings of depression (OR: 2.7; 95%CI: 1.3–5.5) and anxiety (OR: 2.6; 95%CI: 1.2–5.5) in the past 30 days. Just over half (55.3%) reported sexual harassment in more than one place, which was also associated with poor substance use and mental health outcomes.

**Conclusions:**

Findings suggest that sexual harassment experiences are pervasive, occur in girls' immediate and everyday environments, and are associated with girls’ substance use and adverse mental health outcomes.

## Highlights

• We found a high prevalence of recent experiences of sexual harassment (65%) among this clinic-based sample of girls.
• Victimization is largely occurring in girls’ immediate environments and predominantly perpetrated by strangers.
• Sexual harassment was associated with substance use, as well as depression and anxiety among adolescent girls.

## 1 Introduction

Sexual harassment, defined as unwanted and unwelcome behavior of a sexual nature, is a form of sexual violence that is extremely prevalent among youth (Chiodo, Wolfe, Crooks, Hughes, & Jaffe, 2009; Clear et al., 2014; Hill & Kearl, 2011). Young women and girls in particular are more likely to experience sexual harassment than their male counterparts (Fairchild & Rudman, 2008; Mitchell, Ybarra, & Korchmaros, 2014; Turner, Finkelhor, Hamby, Shattuck, & Ormrod, 2011). A large nationally conducted study among students in grades 7 through 12 examining the prevalence of sexual harassment experienced within the past year found that more than half (52%) of girls reported being sexually harassed, compared with 35% of their male peers (Hill & Kearl, 2011). Of concern, there is increasing evidence from neighborhood studies that prevalence estimates are even greater and frequency of exposure is higher among young women living in high-poverty neighborhoods, where other forms of sexual violence and community violence are high and often intersect (Bureau of Justice Statistics, 2017; Popkin, Leventhal, & Weismann, 2010; Reed, Silverman, Raj, Decker, & Miller, 2011; Smith, Gallagher, Popkin, Mireles, & George, 2014). Specifically, previous studies found that high levels of community violence are associated with partner and sexual victimization of women and girls (Reed et al., 2011). Thus, more research is needed to assess the prevalence of sexual harassment among adolescent girls residing in such communities where multiple forms of violence are occurring in high proportions.

Sexual harassment is important to study because it not only carries social implications as a form of gender discrimination and sexual aggression but it also poses a serious public health concern for adolescent girls in the United States and worldwide (Fairchild & Rudman, 2008; Lahsaeizadeh & Yousefinejad, 2012; Madan & Nalla, 2016; Nahar, 2013; Neupane & Chesney-Lind, 2014). The detrimental effects of sexual harassment on mental health outcomes have been well-documented (Exner-Cortens, Eckenrode, & Rothman, 2013; Hand & Sanchez, 2000; Lee, Croninger, Linn, & Chen, 1996). Adolescent girls who have experienced sexual harassment report higher rates of depression and anxiety (Bucchianeri, Eisenberg, Wall, Piran, & Neumark-Sztainer, 2014; Gruber & Fineran, 2007, 2008). Several studies have also documented significant associations between sexual harassment and substance use (i.e., alcohol and drugs) among adolescent girls (Bucchianeri et al., 2014; Gruber & Fineran, 2007; Gruber & Fineran, 2007). However, many previous studies examining sexual harassment in relation to mental health and substance use have not focused on adolescent girls who may be at greatest risk, particularly those residing in low-income/high-poverty communities. The prevalence may likely be greater in these communities and the implications on mental health and substance use may be more concerning, especially if the frequency of exposure is greater.

Few studies have sought to examine the full range of perpetrators, types of harassment, and places in which girls experience harassment. Many studies on sexual harassment among adolescent girls have either not asked about the location of the event or focused specifically on sexual harassment occurring at school (Espelage, Hong, Rinehart, & Doshi, 2016; Hill & Kearl, 2011; Lee et al., 1996; Lichty & Campbell, 2012), at work (Fineran & Gruber, 2009; McGinley, Richman, & Rospenda, 2011; Rauscher, 2008), and in the neighborhood (Popkin et al., 2010; Smith et al., 2014). Thus, little is known regarding where reported sexual harassment is most commonly experienced among girls, across various public places (e.g., neighborhood, work, school), and the perpetrators of such harassment. In addition, more research is needed to assess how many girls are reporting experiencing sexual harassment in multiple places (i.e., at school, on the bus, in their neighborhoods) and associated adverse health outcomes, such as substance use and mental health outcomes.

In summary, future research on sexual harassment among adolescent girls is needed that focuses on those residing in low-income/high-poverty communities, assesses sexual harassment experiences that occur across various places, identifies the perpetrators involved, and examines associations with poor mental health and substance use outcomes. This study aims to address the current gaps in the literature by 1) assessing experiences of sexual harassment among adolescent girls by type (e.g., gestures, unwanted touching), place (e.g., school, neighborhood), and perpetrator (e.g., peer, stranger); 2) assessing girls’ experiences of sexual harassment in relation to substance use and poor mental health outcomes; and 3) assessing the proportion of girls reporting experiences of sexual harassment in *more than one context* and relation to substance use and mental health outcomes.

## 2 Methods

### 2.1 Study design

The current study involves cross-sectional data on sexual harassment and substance use and mental health outcomes, collected as part of a larger study on adolescent girls’ spatial mobility and risk for sexually transmitted infections (STIs) in a low-resource community on the US side of the California-Mexico border. More than one-quarter of residents in the study neighborhood live below the national federal poverty line (Carmen, Proctor, and Smith 2008). High rates of drug-related crime and violence, including sexual violence, have been widely documented in the border region and have remained high over time (Jones, 2011; Oustaunau & Bane, 1999; Shirk, 2010).

Participants were recruited from an adolescent health clinic, where clinic providers and research staff from the study approached them in the waiting room. In order to be eligible for the study, participants had to: a) be female; b) be aged 15–19 years; c) report being sexually active in the past 6 months (given that the focus of the larger study was on STI risk); d) speak English; and e) be able to provide consent. Of the participants who were approached and eligible (n = 182), 87% (n = 159) participated. The most common reason for nonparticipation was lack of time. All participants responded to survey questions on sexual harassment and were included in our analyses (n = 159).

### 2.2 Data collection procedures

Upon establishing eligibility, participants completed the informed consent procedures administered by research assistants. Participants then completed a 45- to 60-min self-guided questionnaire. The questionnaire was administered on tablets and collected general information on sociodemographic factors (age, ethnicity, education, employment, relationship status, modes of transportation), sexual and reproductive health (STI history, previous pregnancies, use of sexual health services, contraception), sexual behavior (number of partners, condom use by type of partner), alcohol/substance use (lifetime use, use in past 30 days, frequency of use), mental health (depression, anxiety, suicidal ideation), experiences of violence and harassment (sexual coercion/sexual violence, physical violence, sexual harassment in public), and social media use. Participants received a $20 gift card upon completion of the survey. The Institutional Review Board (IRB) at the University of California, San Diego (UCSD) approved this study.

### 2.3 Measures

Sexual harassment. Participants were asked about various experiences of sexual harassment that occurred in the past 6 months, including harassment type, places where the harassment occurred, and perpetrators of the harassment. *Sexual harassment victimization* was assessed using items from the American Association of University Women (AAUW) survey (AAUW, 2001). These 4 items asked participants whether a man/boy or group of men/boys have, in the past 6 months: a) made unwanted sexual comments, jokes, or gestures towards them in public; b) exposed themselves sexually in public; c) touched, grabbed, or pinched them in a sexual way that they did not want; and/or d) touched them with any part of their body—including getting too close or rubbing up against them—when they did not want this. We summated responses and created a dichotomized variable indicating any sexual harassment in the past 6 months versus no sexual harassment in the past 6 months, as done in the original AAUW study (AAUW, 2001). This served as the primary independent variable of interest. The measure has a Cronbach's alpha of .56, which suggests low internal reliability (DeVellis, 2012, pp. 109–110); the low alpha may be due to the small number of items, given that individual items were highly associated with each other (all *P* values < 0.03), which were not at the level of collinearity but precluded assessment of their independent associations with our outcomes of interest.

Based on prior research documenting that sexual harassment occurs across diverse locations in public and in schools (Crouch, 2009; Espelage et al., 2016) as well as formative qualitative research with youth and providers in preparation for this study, we expanded the AAUW (2001) measure to include an additional item assessing the *locations of sexual harassment*. The item asked: “In the past, when boys or men have done any of these things to you, where did it happen?” Responses were “at school,” “at home,” “at work,” “on public transportation,” “my neighborhood,” “another neighborhood nearby,” or “other place,” and responses were not mutually exclusive. Participants were able to write in any “other place” they experienced sexual harassment. Our qualitative research that guided this question highlighted that sexual harassment across diverse locations was indicative of the behavior as normative in the girls’ social environment. We developed a variable that represented whether participants reported sexual harassment in more than one place in the previous 6 months (yes/no). This also served as an independent variable of focus.

Again, based on our formative research, we found that girls were often familiar with those who sexually harass them, and some are even viewed as friends. Thus, we created for this survey an additional item on relationship to the perpetrator assessed for the subsample reporting sexual harassment. We asked, “When boys or men have done any of these things to you, who did this?” Responses were: “boys/men I did not know,” “boys/men I knew of or have seen but am not friendly with,” and “boys/men I knew pretty well or who are my friends.” Participants could choose more than one response (i.e., check all that apply). This sexual harassment variable was used for descriptive purposes to understand the nature of the harassment experienced.

Substance use. To assess drug use behaviors, we asked about lifetime use and use in the past 30 days of marijuana and 11 different substances, including cocaine, stimulants, and prescription drugs, using items adapted from the Texas Christian University Drug Screen 2 (TCU-DS2) (Knight, Simpson, & Hiller, 2002, pp. 259–272) and the 10-question Alcohol Use Disorders Identification Test (AUDIT-10) (Babor, 2001). For each substance, we asked about lifetime use and use in the past days among those reporting yes to lifetime use. Using the item on marijuana use, we created a variable of whether they had used *marijuana within the past 30 days* (yes/no). Separately, we summated responses on the other 11 drugs and created a dichotomized variable of *other drug use within the past 30 days* (yes/no).

Alcohol Use. We included three items: taken from the AUDIT-10 to measure lifetime alcohol use and use in the past 30 days, (Babor, 2001). We asked participants whether they had a) *ever drank an alcoholic beverage* and b) *drank an alcoholic beverage in the past 30 days* (yes/no). Participants who reported alcohol use in the past 30 days were then asked about *binge drinking* with the one item measure: “How often in the past 30 days did you have 5 or more alcoholic drinks in one occasion?” on a 0 (*never*) to 5 (*everyday)* Likert-type scale. A variable was created to reflect participants who reported any binge drinking in the past 30 days. Based on our qualitative assessments with this population to refine survey measures, we asked about 5 drinks in one occasion rather than 4 drinks, which is typically used to define binge drinking among girls (CDC, 2013).

Mental health. Single-item measures were used to ask participants whether they felt a) *worried, tense, or anxious in the past 30 days* (Spitzer, Kroenke, Williams, & Lowe, 2006) and/or b) *felt down, depressed, or hopeless in the past 30 days* (Kroenke, Spitzer, & Williams, 2001), using a Likert-type scale of 1 *(not at all*) to 4 (*nearly every day*). A variable was created to reflect participants who reported feeling anxious or depressed at all in the previous 30 days.

Demographics. Demographic variables such as age, race (e.g., identifying as Hispanic/Latina), and living situation (e.g., living with parents) were used to characterize the sample. Except age, all demographic variables were categorical.

### 2.4 Data analysis

Descriptive statistics were used to summarize girls' demographic characteristics, types of sexual harassment, perpetrators involved, and the places in which these events occur. A t-test and chi-square and Fisher's exact tests were used to examine: 1) the association of demographic variables with any sexual harassment in the past 6 months and 2) sexual harassment in relation to each study outcome, including participants' alcohol use in the past 30 days, drinking 5 or more alcoholic drinks at once in the past 30 days, marijuana use in the past 30 days, other drug use ever, feeling depressed in the past 30 days, and feeling anxious in the past 30 days. Crude and adjusted logistic regression/exact conditional logistic regression models were used to examine sexual harassment (and experiences of sexual harassment in more than one place) in relation to each outcome. Demographic variables associated with any outcome at *P* < .05 were included in all adjusted models. Findings from logistic regression models were presented as odds ratios (ORs) and exact ORs with associated 95% confidence intervals (CIs), and significance of individual variables was evaluated using Wald chi-square and exact conditional tests. All analyses were conducted using SAS® version 9.4.

## 3 Results

### 3.1 Sample characteristics

Table 1 presents the sociodemographic characteristics and prevalence of sexual harassment for the full study sample (*N* = 159). The average age of the sample was 17 years. Most participants identified as Latina (76.7%), were born in the United States (77.2%), and lived with their parents (83.5%).

**Table 1:** Sociodemographic characteristics and bivariate analysis by experiences of any type of sexual harassment in the past 6 months among adolescent girls aged 15–19 years (*N* = 159)^a^.

Demographic characteristics		Reported any sexual harassment (in the past 6 months)				Reported any sexual harassment in more than one location (in past 6 months)			
		Total (N = 159) n (%)	Yes (n = 104) n (%)	No (n = 55) n (%)	*X*^2^, (*P* value)	Total (N = 103^b^) n (%)	Yes (n = 57) n (%)	No (n = 46) n (%)	*X*^2^, (*P* value)
Age^c^	17.05 (1.08)^c^		16.99 (1.04)^c^	17.16 (1.15)^c^	0.96^b^ (0.34)	16.98 (1.04)^c^	17.05 (1.09) a	16.89 (0.97)^c^	-0.78^d^ (0.43)
Race/Ethnicity					<.0001^d^ (0.0608)				0.0002^e^ (0.158)
White	23 (14.5)		17 (16.4)	6 (10.9)		17 (16.5)	8 (14.0)	9 (19.6)	
Asian	22 (13.8)		18 (17.3)	4 (7.3)		18 (17.5)	14 (24.6)	4 (8.7)	
AI/NH	11 (6.9)		5 (4.8)	6 (10.9)		5 (4.85)	3 (5.3)	2 (4.3)	
Black or African American	5 (3.2)		5 (4.8)	0 (0.0)		5 (4.85)	4 (7.0)	1 (2.2)	
Other^f^	98 (61.6)		59 (56.7)	39 (70.9)		58(56.3)	28 (49.1)	30 (65.2)	
Latino					3.58 (0.058)				12.28 (0.0005)
Yes	122 (76.7)		75 (72.1)	47 (85.4)		74 (71.8)	33 (57.9)	41 (89.1)	
No	37 (23.3)		29 (27.9)	8 (14.6)		29 (28.2)	24 (42.1)	5 (10.9)	
Born in the US					0.04 (0.83)				6.48 (0.01)
Yes	122 (77.2)		79 (76.7)	43 (78.2)		79 (76.7)	49 (86.0)	29 (64.4)	
No	36 (22.8)		24 (23.3)	12 (21.8)		24 (23.3)	8 (14.0)	16 (35.6)	
Live with parents					1.98 (0.16)				1.02 (0.31)
Yes	132 (83.5)		90 (86.5)	42 (77.8)		89 (86.4)	51 (89.5)	38 (82.6)	
No	26 (16.5)		14 (13.5)	12 (22.2)		14 (13.6)	6 (10.5)	8 (17.4)	

*Abbreviations:* AI, American Indian; NH, Native Hawaiian.

*Notes*.

a Some column and row values may not always add up because of missing data.

b Among 104 participants who reported experiencing sexual harassment, only 103 reported the number of locations in which they had these experiences.

c Mean (SD).

d T-value, using t-test.

e Total probability, using Fisher's exact test.

f Other category mostly represents participants who considered themselves Latino (n = 72).

Of the 159 adolescent girls, 65% (n = 104) reported experiencing any sexual harassment during the past 6 months. Among those experiencing sexual harassment in the past 6 months, 55.3% (n = 57) experienced sexual harassment in more than one location. There were no statistically significant differences in experiencing sexual harassment by demographic variables. However, among those who experienced any sexual harassment, Latina adolescents appeared less likely to report experiencing sexual harassment in more than one location (44.6%) than non-Latinas (82.8%) (*X*^2^ 12.3; *P* = .0005). In addition, among those experiencing sexual harassment, those born in the United States (62.0%) were more likely to report sexual harassment in more than one place than those not born in the United States (33.3%) (*X*^2^ = 6.5; *P* = 0.01).

### 3.2 Sexual harassment type, place, and perpetrators

The most frequent type of sexual harassment experienced involved sexual comments, jokes, or gestures (57.2%), followed by being touched with any part of a perpetrator's body in an unwanted sexual way (12.6%), being touched, grabbed, or pinched in a sexual way (12.6%), and experiences with men exposing themselves sexually in public (3.8%). More than 30% of girls reported experiencing two or more types of sexual harassment during the past 6 months (data not shown in tables). Girls reported that, for the most part, harassment took place on public transportation (50.5%), at school (42.7%), and in their or surrounding neighborhoods (39.8%). In addition, 5.8% of girls reported experiencing sexual harassment at work, 3.0% reported experiencing sexual harassment at home, and another 41.8% reported experiencing sexual harassment in some other place. More than half (55.3%) of the girls who experienced sexual harassment reported experiencing it in more than one place; 18.4% reported experiencing sexual harassment in 2 places, 20.4% in 3 places, 9.7% in 4 places, and 6.8% in 5 or more places. The perpetrators of sexual harassment were predominantly boys/men who were *not* known to the girls (82.7%).

### 3.3 Substance use and mental health outcomes

Less than half (38.3%) of participants reported alcohol use or binge drinking in the past 30 days. Slightly more than one-third (34.4%) reported marijuana use in the past 30 days. Slightly less than one-quarter (22.6%) reported having ever used drugs. High proportions reported experiencing depression (60.1%) and anxiety (73.9%) in the past 30 days. (Table 2).

**Table 2:** Crude and adjusted logistic regression findings: The relation between reporting experiences of sexual harassment (exposure) in past 6 months and mental health and substance abuse (outcomes).

Variable	Reported any sexual harassment (in the past 6 months)					Reported any sexual harassment in more than one location (past 6 months)				
	Total (*N* = 159) n (%)	Yes (n = 104) n (%)^c^	No (n = 55) n (%)^c^	Crude OR (95% CI)	Adjusted OR (95% CI)^a^	Total (*N* = 103^b^) n (%)	Yes (n = 57) n (%)	No (n = 46) n (%)	Crude OR (95% CI)	Adjusted OR (95% CI)^a^
Alcohol use in past 30 days										
No	95 (61.7)	52 (51.0)	43 (82.7)	Ref	Ref	52(51)	24(42.1)	28(62.2)	Ref	Ref
Yes	59 (38.3)	50 (49.0)	9 (17.3)	4.6 (2.0–10.4)^├^	4.5 (2.0–10.2)^├^	50(49)	33(57.9)	17(37.8)	2.3 (1.1–5.1)*	2.5 (1.1–5.8)*

Binge drinking in past 30 days										
No	92 (61.7)	52 (52.0)	40 (81.6)	Ref	Ref	51(51.5)	25(45.5)	26(59.1)	Ref	Ref
Yes	57 (38.3)	48 (48.0)	9 (18.4)	4.1 (1.8–9.3) ^├^	4.2 (1.8–9.8)^├^	48(48.5)	30(54.6)	18(40.9)	1.7 (0.8–3.9)	1.9 (0.8–4.5)
Marijuana use in past 30 days										
No	101 (65.6)	60 (58.8)	41 (78.9)	Ref	Ref	60(58.8)	31(54.4)	29(64.4)	Ref	Ref
Yes	53 (34.4)	42 (41.2)	11 (21.1)	2.6 (1.2–5.7)*	2.6 (1.2–5.7)*	42(41.2)	26(45.6)	16(35.6)	1.5 (0.7–3.4)	1.5 (0.6–3.5)
Lifetime drug use										
No	120 (77.4)	71 (69.6)	49 (92.4)	Ref	Ref	71(69.6)	34(59.7)	37(82.2)	Ref	Ref
Yes	35 (22.6)	31 (31.4)	4 (7.6)	5.3 (1.8–16.1)**	5.4 (1.8–16.4)**	31(30.4)	23(40.4)	8(17.8)	3.1 (1.2–7.9)*	3.2 (1.2–8.6)*
Feeling depressed in past 30 days										
No	61 (39.9)	31 (30.7)	30 (57.7)	Ref	Ref	31(31)	14(25.5)	17(37.8)	Ref	Ref
Yes	92 (60.1)	70 (69.3)	22 (42.3)	3.1 (1.5–6.2)**	2.7 (1.3–5.5)**	69(69)	41(74.6)	28(62.2)	1.8 (0.8–4.2)	1.3 (0.5–3.3)
Feeling anxiety in past 30 days										
No	40 (26.1)	19 (18.8)	21 (40.4)	Ref	Ref	19(19)	5(9.1)	14(31.1)	Ref	Ref
Yes	113 (73.9)	82 (81.2)	31 (60.6)	2.9 (1.4–6.2)**	2.6 (1.2–5.5)*	81(81)	50(90.9)	31(68.9)	4.5 (1.5–13.8)**	3.5 (1.1–11.4)*

Abbreviations: CI, confidence interval; OR, odds ratio.

Notes.

*P < .05, **P < .01, ^├^*P* < 0.001.

a Adjusted ORs are controlled for age and Latino status.

b Among 104 participants who reported experiencing sexual harassment, only 103 reported the number of locations in which they had these experiences.

c Column percentages.

### 3.4 Associations between experiences with sexual harassment with substance use and mental health outcomes

Adjusted logistic regression models show that, compared with girls who had not been harassed, girls who experienced any type of sexual harassment during the past 6 months had statistically significantly greater odds of alcohol use (OR: 4.5; 95% CI: 2.0–10.2), binge drinking (OR: 4.2; 95% CI: 1.8–9.8), and marijuana use (OR: 2.6; 95% CI: 1.2–5.7) within the past 30 days (Table 2). Girls who reported experiencing sexual harassment also had greater odds of reporting lifetime drug use (OR: 5.4; 95% CI: 1.8–16.4) than those not harassed. Results also indicate that adolescent girls who reported sexual harassment had significantly greater odds of feeling depressed (OR: 2.7; 95% CI: 1.3–5.5) and anxious (OR: 2.6, 95% CI: 1.2–5.5) within the past 30 days than those who did not report harassment.

Experiencing sexual harassment in more than one location was also associated with alcohol use in the past 30 days (OR: 2.5; 95% CI:1.1–5.8), ever using drugs (OR: 3.2; 95% CI: 1.2–8.6), and feeling anxious in the past 30 days (OR: 3.5; 95% CI: 1.1–11.4). Notably, we also found evidence of a dose-response relationship when looking at the number of places girls’ experience sexual harassment (as a continuous variable) and relation to alcohol use, drug use, and feeling anxious in the past 30 days. Although not statistically significant, trends were observed when looking at other study outcomes as well but were likely limited because of the small sample size.

## 4 Discussion

The current study documents that this urban, predominantly racial/ethnic minority sample of girls is experiencing a very high rate of sexual harassment, with almost two-thirds of girls (65.4%) reporting harassment in just the past 6 months. We found that victimization largely occurrs in public spaces, including on public transportation and at school, and is predominantly perpetrated by strangers. Findings also demonstrate that sexual harassment is associated with increased risk for substance use, depression, and anxiety among adolescent girls from a low-income, predominantly Hispanic community near the US-Mexico border. Our study builds on previous research by documenting the multiple settings and perpetrators that characterize girls’ experiences of sexual harassment, reporting the proportion of girls who experience sexual harassment in more than one setting and its relation to poor health outcomes, and focusing these assessments on a sample of adolescent girls from a community where violence, including sexual violence and harassment, occurs in high proportions.

The proportion of girls who reported experiencing sexual harassment (65% in the past 6 months) among this sample of girls aged 15–19 years was higher than those reported in many other studies (Mitchell et al., 2014; Fairchild & Rudman, 2008). Many studies have restricted assessments of sexual harassment experiences among adolescent girls to those that occur specifically in school settings (e.g., Chiodo et al., 2009). In contrast, our study asked about experiences of sexual harassment in multiple places. The high proportion of sexual harassment experiences reported in our study is aligned with previous findings that young women living in high-poverty neighborhoods may be at especially high risk for experiencing sexual harassment in their neighborhoods (Briggs, Popkin, & Goering, 2010; Popkin et al., 2010; Smith et al., 2014). Our study builds on this previous research by suggesting that girls in high-poverty neighborhoods not only experience high levels of sexual harassment in their neighborhoods but may also experience high levels at school and on public transportation. Additionally, of the previous studies that were not limited to one setting (e.g., school), most reported that the majority of girls experienced sexual harassment (e.g., Eom, Restaino, Perkins, Neveln, & Harrington, 2015); however, most studies assessed lifetime experiences, whereas our study reported recent experiences (in the previous 6 months).

Notably, we found some differences in experiences of sexual harassment by sociodemographic variables: Latina and non–US-born adolescent girls were less likely to report experiencing sexual harassment in more than one location than non-Latina and US-born adolescents. Although more research is needed to understand these findings, adolescent girls who are recent immigrants may be more likely to go to places with their families (rather than alone), making them less exposed to sexual harassment. In our study, girls who were non-Latina were predominantly Filipina—and in a predominantly Latina community, Filipina girls may be considered the minority, which may put them at greater risk for being targeted for harassment, including sexual harassment. More research is needed to further assess and better understand these findings.

Experiencing sexual harassment at school has been shown to compromise girls' perceptions of safety at school (Chiodo et al., 2009) and is linked to poor academic outcomes (Lichty & Campbell, 2012); our study also documents that high proportions of girls experience sexual harassment in multiple immediate and everyday environments in addition to school, including on public transportation and in their neighborhoods. Slightly more than half of girls reported experiencing sexual harassment on public transportation and almost half reported it in their neighborhoods, which may impede girls’ mobility (e.g., freedom of movement) through fear of harassment and, in turn, uncertainty of their safety in these settings (Loukaitou-Sideris, 2008). Previous studies that have highlighted the concern of sexual harassment on public transport have often been conducted in non-US settings, including multi-country comparisons using national data (Gekoski, Gray, Adler, & Horvath, 2017). Our study findings suggest that more research on sexual harassment in various public places, including public transport, is needed in the United States, particularly within urban settings among young women and girls. Additionally, almost half (42%) of our sample reported experiencing sexual harassment in a place other than school, the neighborhood, public transport, work, or home; thus, future research is needed to identify the wide range of places in which girls are experiencing sexual harassment.

Consistent with other studies (Bucchianeri et al., 2014; Goldstein, Malanchuk, Davis-Kean, & Eccles, 2007; Gruber & Fineran, 2007), our findings show that sexual harassment was associated with adverse substance use and mental health outcomes among high school girls. New to the literature, we found that the number of places in which girls experience sexual harassment is also associated with adverse substance use and mental health outcomes. Our findings suggest that the pervasiveness of sexual harassment in girls' environments should considered when designing intervention strategies. Previous research has also found that chronic experiences of sexual harassment are linked with poor substance use and mental health outcomes (McGinley, Wolff, Rospenda, Liu, & Richman, 2016). Thus, future research is needed to investigate both the frequency and the number of locations in which sexual harassment occurs, to better understand how each of these may impact girls' well-being (i.e., on a typical day, are girls experiencing sexual harassment in their neighborhoods on their way to school, at school, and on public transport to and from school?). Most of the sexual harassment that girls are experiencing is likely chronic and occurring in various places. Thus, using current measures, we may not be capturing the full extent of girls’ experiences of sexual harassment.

Perpetration of sexual harassment was largely by those unknown to female participants, which is aligned with our finding that the majority of sexual harassment was reported in public places, such as public transport and neighborhoods. In contrast, previous studies have found that sexual harassment occurring in school is often perpetrated by those known to the victim (Espelage et al., 2016). Thus, future work is needed that considers a broad range of places, to measure the perpetrators of sexual harassment by place in which harassment occurs. Increasingly, adolescent girls are also experiencing sexual harassment online. (Henry & Powell, 2018). Given that a substantial number of participants reported sexual harassment as occurring in “other” places (other than those we listed in the survey), it is possible that one of these places may be online. Future measures may need to encompass experiences in person and online and capture the unique set of experiences that occur in person versus online (Henry & Powell, 2015). Depending on whether incidents are perpetrated in person or online, the frequency and most common perpetrators of these will likely differ as well. For example, research on cyber sexual harassment has often found that relationship partners are also perpetrators of this harassment (e.g., pressuring girls via digital mediums to send sexual photos or to do something sexual) (Henry & Powell, 2018). Many in-person sexual harassment measures do not include being pressured to do something sexual in person, and few studies have measured sexual pressure perpetrated by intimate partners as a form of sexual harassment (e.g., asking participants if anyone has ever pressured them to engage in sexual activity when they were not ready or did not want to, including relationship partners as possible perpetrators). Asking a participant about past sexual pressure/coercion would capture more comprehensive information regarding incidents of sexual pressure/coercion than only asking if a participant has ever had sex *as a result* of being pressured or coerced, which is more commonly measured in sexual violence studies. Thus, future measures of sexual harassment may need to broaden their definition to include sexual pressure/coercion and assess relationship and sexual partners as possible perpetrators. Unfortunately, our measure of perpetrators was limited in that we could not assess whether perpetrator characteristics vary by the type of sexual harassment reported or the place in which it occurred. In addition to assessing the relationship of the perpetrator to the victim (including whether the perpetrator was known or unknown to the victim, as well as perpetration by sexual and dating partners), future research may also benefit from assessing the sociodemographic characteristics of the perpetrator (i.e., was the perpetrator a similar age, sex, or race/ethnicity as the victim?).

### 4.1 Limitations

Although our findings offer important insight into the role of sexual harassment and its potential health consequences for adolescent girls, they must be considered in light of certain limitations. The study relies on self-reported data and is subject to recall and social desirability biases. To reduce recall bias, we focused on recent experiences, from the past 30 days to 1 year, and to reduce social desirability bias, we used self-administered surveys (Krumpal, 2013). Future longitudinal study with larger samples will be needed to establish the temporality of associations and to better understand the unique effects of different types of sexual harassment, including distinct effects based on perpetrator type, place in which the harassment occurred, and frequency of harassment. The current study had a small sample size and was not originally powered for the current research question; however, findings were statistically significant, despite the likely underreported outcomes. The study also has limited generalizability to sexually active adolescent girls recruited from a single health center serving an underserved, predominantly Hispanic community.

### 4.2 Conclusion

Despite limitations, our findings suggest that sexual harassment is pervasive, occurs in adolescent girls' immediate environments (e.g., school, neighborhood), and is associated with adverse substance use and mental health outcomes. Our findings suggest that experiences of sexual harassment create a stressful environment and lead to poor health-related outcomes for girls. Given that most girls in our study have experienced sexual harassment and its link to poor health outcomes, sexual harassment should be considered a major public health concern. Our findings suggest that sexual harassment is not only pervasive but has similar health outcomes as other forms of sexual violence (e.g., sexual assault, sexual coercion) experienced by adolescent girls (CDC, 2017). The interconnections of sexual harassment and other forms of sexual violence suggest that prevention efforts may benefit from addressing the root causes across these forms of violence, including social determinants (e.g., poverty, racism) and harmful gender norms related to the sexual objectification of girls and that promote women's subservience to men (Reed et al., 2011). Furthermore, sexual harassment, like other forms of gender-based violence against girls and women, is not only a health concern but an issue of human rights, with evidence that it interferes with girls' mobility, opportunities, safety, and overall well-being.

## Conflict of interest disclosures

Authors do not have any potential conflicts of interest or financial disclosures to report.

## Funding

This research was funded by the Eunice Kennedy Shriver National Institute on Child Health and Development (R21 HD073610; Principal Investigators: Reed and Raj). Elizabeth Reed was also supported by the National Institute of Mental Health (K01MH099969, Principal Investigator: Reed). Marissa Salazar was supported by the National Institute of Drug Abuse (T32: DA023356, Principal Investigator: Strathdee).

## Ethical statement

The Institutional Review Board (IRB) at the University of California, San Diego (UCSD) approved this study.
